# Nano-scaled emulsion and nanogel containing *Mentha pulegium* essential oil: cytotoxicity on human melanoma cells and effects on apoptosis regulator genes

**DOI:** 10.1186/s12906-023-03834-y

**Published:** 2023-01-09

**Authors:** Sareh Azadi, Mahmoud Osanloo, Elham Zarenezhad, Mojtaba Farjam, Akram Jalali, Ali Ghanbariasad

**Affiliations:** 1 Department of Medical Biotechnology, School of Advanced Technologies in Medicine, Fasa University of Medical Sciences, Fasa, Iran; 2 Department of Medical Nanotechnology, School of Advanced Technologies in Medicine, Fasa University of Medical Sciences, Fasa, Iran; 3 Noncommunicable Diseases Research Center, Fasa University of Medical Sciences, Fasa, Iran; 4 Department of Pharmacology, School of Medicine, Fasa University of Medical Sciences, Fasa, Iran; 5 Research Center for Molecular Medicine, Hamadan University of Medical Sciences, Hamadan, Iran

**Keywords:** Skin Cancer, Nanotechnology, Apoptosis, Topical drug delivery

## Abstract

**Background:**

Topical drug delivery using nanoemulsions and nanogels is a promising approach to treating skin disorders such as melanoma.

**Methods:**

In this study, the chemical composition of *Mentha pulegium* essential oil with five major compounds, including pulegone (68.11%), l-menthone (8.83%), limonene (2.90%), iso-pulegone (2.69%), and iso-menthone (1.48%) was first identified using GC-MS (Gas chromatography–Mass Spectrometry) analysis. Afterward, a nano-scaled emulsion containing the essential oil with a droplet size of 7.70 ± 1 nm was prepared. Nanogel containing the essential oil was then prepared by adding (2% w/v) carboxymethyl cellulose to the nano-scaled emulsion. Moreover, the successful loading of *M. pulegium* essential oil in the nano-scaled emulsion and nanogel was confirmed using ATR-FTIR (Attenuated total reflectance-Fourier Transform InfraRed) analysis. Then, human A375 melanoma cells were treated with different concentrations of samples, the MTT assay evaluated cell viability, and cell apoptosis was confirmed by flow cytometry. In addition, the expression of apoptotic and anti-apoptotic genes, including Bax and Bcl-2, was evaluated using the qPCR (quantitative Polymerase Chain Reaction) technique.

**Results:**

The results showed that cell viability was reduced by 90 and 45% after treatment with 300 μg/mL of the nanogel and nano-scaled emulsion. As confirmed by flow cytometry, this effect was mediated by apoptosis. Furthermore, gene expression analysis showed up-regulation of Bax and down-regulation of Bcl-2 genes. Therefore, the prepared nanogel, with high efficacy, could be considered a potent anticancer agent for supplementary medicine and in vivo research.

## Introduction

Cancer was the cause of about 10 million deaths in 2020, and skin cancer, with around 1.3 million cases, was the fifth most common type worldwide [1]. Non-melanoma and melanoma are the main classes of skin cancer. Non-melanoma skin cancer originates from the epidermis’s keratinocytes, and melanoma originates from melanocytes at the deepest layer of the epidermis [2, 3]. Melanoma is responsible for 80% of mortality cases in skin cancer. Chronic ultraviolet exposure, fair skin, blond hair, congenital moles, suppressed immune systems, genetic polymorphisms, alcohol consumption, genetic factors, and family history are risk factors for melanoma [4]. In addition, surgery, chemotherapy, and radiotherapy as common treatment approaches possess various side effects. For instance, doxorubicin has high drug efficacy against many cancers. However, it has serious side effects, such as heart damage [5].

Essential oils (EOs) are lipophilic substances secreted as secondary metabolites from different parts of aromatic plants. They are a great source of potent anticancer compounds [6, 7]. EOs have many biological properties, including antibacterial, antiviral, anti-inflammatory, anti-fungal, anti-mutagenic, anti-carcinogenic, and anti-oxidant effects [8, 9]. However, their practical usage has been questioned as having low efficacy compared to synthetic drugs. In addition, the biological activity of EOs is lost through volatilization or degradation by high temperatures, oxidation, and UV light [10, 11]. In the last decade, preparing nanoformulations containing EOs received much attention as a promising solution to meet the challenges. Nanoformulation dosage forms with different states, including liquid (emulsions, micelles, and liquid solutions), semi-liquid (gels, cream, and liposome), and solid (solid lipid nanoparticles, nanocapsules, and nanospheres) which have been proposed for protecting EOs from the external environment and improving their efficacy [12, 13]. Nano-scaled emulsions and nanogels with high biocompatibility, biodegradability, and proper stability are promising formulations in topical drug delivery [14, 15].

Mentha is a well-known genus of medicinal plants; they belong to the Lamiaceae family and include 25–30 species. Mentha spp. are widely cultivated in various parts of the world, such as Asia, Europe, North Africa, and North America [16, 17]. Cytotoxic effects of many species of Mentha have been reported in the literature. For instance, *M. longifolia* EO has been reported against two human breast cancer cell lines, MDA-MB-468 and MCF-7 (IC50 values not reported) [18]. However, the IC50 value of *M. arvensis* EO against the thyroid cancer cell line (HTh-7) was reported at 0.6 μL/mL [19]. Besides, the cytotoxic effects (IC50 values) of *M. spicata* EO against human colon cancer (HCT-116), human breast adenocarcinoma (MCF-7), and human ductal carcinoma (T47D) cell lines were reported at 279, 975, and 324 μg/mL [20]. Moreover, the anticancer effect of *M. piperita* EO against MCF-7 (IC50: 165 μg/mL), MDA-MB-231 (IC50: 25 μg/mL), MDA-MB-468 (IC50: 2536 μg/mL), HepG-2 liver cancer cells (IC50 not reported), HCT-116 colorectal cancer cells (IC50: 500 μg/mL), K-562 leukemia cells (IC50: 16 μg/mL), SGC-7901 gastric cancer cells (IC50: 39 μg/mL), and A-549 lung cancer cells (IC50 not reported) were reported [21–24]. Furthermore, *M. pulegium* is another species of the Mentha family; it has traditionally been used for skin diseases, colds, digestive and liver disorders, and cosmetics [25, 26]. Moreover, its IC50 values on human breast and prostate cancer cells (MCF-7 and PC3) were reported at 80 and 98 μg/mL [27].

Herein, for the first time, a comparison was made between the cytotoxic effects of *M. pulegium* EO (MPEO) and its nano-scaled emulsion and nanogels dosage forms on A375 human melanoma cells. After that, their effect on inducing apoptosis and expression of apoptotic and anti-apoptotic genes (Bax and Bcl-2) was investigated using flow cytometry and qPCR techniques.

## Materials and methods

### Materials

Bark-extracted MPEO was obtained from Giahessence pharmaceutical Co. (Iran). A375 human melanoma cells (ATCC® CRL-1619) was bought from Pasteur Institute Cell Bank (Tehran, Iran). RPMI (Roswell Park Memorial Institute 1640) medium, fetal bovine serum, and trypsin were purchased from Gibco (BRL, MD, USA). PBS (Phosphate-Buffered Saline), MTT (3-(4.5-Dimethyl-thiazol-2yl)-2.5-diphenyl tetrazolium bromide), DMSO (Dimethylsulfoxide), and *CMC* (Carboxymethyl cellulose) were acquired from Sigma Aldrich (St. Louis, MO, USA). RNA extraction and cDNA synthesis kits were bought from Yectatajiz, Iran. Annexin-V/FITC Apoptosis Detection Kit was purchased from Mab Tag (Germany).

### Chemical constituents of MPEO by GC-MS analysis

MPEO constituents were analyzed by GC-MS analysis using Agilent 6890 gas chromatography connected to 5973 networks mass selective detector. A capillary column of BPX5 (30 m × 0.25 mm, film thickness 0.25 μm) was used to separate MPEO compounds as described in our previous report [15]. The compounds were identified by comparing their mass spectral pattern with the MS libraries (NIST and Wiley) [28, 29].

### Preparation and characterization of nano-scaled emulsion and nanogel

#### Nano-scaled emulsion

Many components of EO are volatile; the spontaneous emulsification manner was thus used to prepare nano-scaled emulsions. This method is developed at room temperature without external energies like ultrasonic or extruder [30]. First, the oil phase was formulated by mixing 6 μL MPEO and 3–24 μL tween 20 using a magnetic stirrer (500 rpm, 10 min). Then, appropriate amounts of PBS were added drop-wisely to reach a final volume (5000 μL) and stirred for 30 min (at 500 rpm).

The prepared nano-scaled emulsions’ droplet size distribution (SPAN) was measured using a dynamic light scattering instrument (scatteroscope, K-ONE NANO. LTD, Korea). SPAN was calculated using D90-D50/D10, where D is the diameter of the droplets, and 10, 50, and 90 are the percentile of droplets with lower diameters than these points. The nano-scaled emulsion with a droplet size of less than 200 nm and SPAN < 1 was considered the optimal sample. It was selected for further investigation, such as the gelation process and biological assays. Furthermore, a blank emulsion was prepared; only MPEO was not used in comparison with the selected nano-scaled emulsion.

Transmission Electron Microscopy (TEM) was carried out to visualize the shape and size of the nano-scaled emulsion droplets. First, a drop of the nano-scaled emulsion dispersion in distilled water was dripped onto a carbon-coated copper grid and dried at room temperature. Then, it was subjected to the TEM device (TEM Philips EM 208S).

#### Nanogel

The nanogel was prepared by adding (2%) CMC powder, as a cross-linker agent, to the nano-scaled emulsion. For hydration of CMC, the mixture was stirred overnight in mild conditions at room temperature (180 rpm). Then, the prepared nanogel was stored at two temperatures (4 and room temperature) for 6 months to investigate its stability. In addition, a blank gel was prepared using a similar process and constituents as the nanogel; only MPEO was not used. Moreover, the viscosity of the nanogel was investigated under atmospheric pressure at 25 °C by a rheometer apparatus (Anton PaarRheometer, model MCR-302, Austria).

The Attenuated Total Reflection-Fourier Transform InfraRed (ATR-FTIR) technique was used to confirm the successful loading of MPEO in the nano-scaled emulsion and nanogel. ATR-FTIR spectra of MPEO, blank emulsion, nano-scaled emulsion, CMC, and nanogel were recorded in the wavelength range of 3500–500 cm^− 1^ (Tensor II – Bruker. Germany).

#### MTT assay

Cells were cultured in RPMI perfect medium (containing 10% fetal bovine serum and 1% penicillin/streptomycin. Cells were grown in a plastic tissue culture T flask (25 cm^2^) at 37 °C with 5% CO2 for experimental assay. The colorimetric MTT assay was used to determine the cytotoxicity of samples against the A375 cells. Briefly, cells were seeded into 96-well plates (10,000 cells/well) and incubated for 24 h. When the cells reached 80% confluence, the wells’ liquid medium was replaced with 100 μL/well of the supplemented RPMI medium. After that, cells were treated with 20, 75, 150, and 300 μg/mL of MPEO (dissolved in PBS containing 0.5% DMSO), blank emulsion, nano-scaled emulsion, blank gel, and nanogel. Incubated plates (24 h) were then treated with 100 μL of MTT solution (5 mg/mL in PBS) and incubated for 4 h. After that, the formazan crystals were dissolved by adding 100 μL/well DMSO. Finally, the wells’ optical density (OD) was read at 570 nm using an ELISA reader (Synergy HTX Multi-Mode Reader, USA). The viability at each concentration was calculated by (OD sample / OD control) × 100.

#### Apoptosis detection using flow cytometry

The flow cytometry technique using Annexin-V/FITC/PI staining was used to confirm the induction of apoptosis. A375 cells (50,000 cells/well) were seeded in 6 well plates. Afterward, they were treated with 300 μg/mL of blank emulsion, nano-scaled emulsion, blank gel, and nanogel and incubated for 24 h at 37 °C. Apoptosis was confirmed using the Annexin-V/FITC/PI Apoptosis Detection Kit protocol (MabTag, GmbH, Germany). Briefly, the cells were harvested by trypsinization, and 5 mL PBS was added. Next, the cells were washed, i.e., the microtube was centrifuged for 5 mins at 200 g, and the supernatant was discarded (2 times). The pellet was finally resuspended in the 1x Annexin-V binding buffer. Then, Annexin-V conjugate and propidium iodide solution were added to the cell suspension and incubated for 20 min in the dark at room temperature. After incubation, 1x binding buffer was added, mixed gently, and analyzed by flow cytometry (BD FACSCalibur, USA). The numbers of viable cells, cells undergoing necrosis (positive for PI), early apoptosis (positive for Annexin-V/FITC), and late apoptosis (double-positive for Annexin-V/FITC and PI) were determined using FlowJo software (BD, Becton, Dickinson and Company).

#### qPCR technique

The expression of apoptotic and anti-apoptotic genes, including Bax and Bcl-2, was evaluated using the qPCR technique. Briefly, A375 cells (50,000 cells/well) were seeded in 6-well plates. They were treated with 300 μg/mL of blank emulsion, nano-scaled emulsion, blank gel, and nanogel and incubated for 24 h at 37 °C. After that, total RNA was extracted using the Trizol RNA extraction kit (Yectatajhiz). Briefly, the liquid content of the wells was discarded and washed with PBS. The cells were then harvested using Trizol and transferred into microtubes. After that, chloroform was added, and after 3 min incubation, microtubes were centrifuged (15,000 rpm) for 15 min at 4 °C. Next, the clear supernatants were transferred to other microtubes, and the same amount of isopropanol was added. Next, microtubes were centrifuged (15,000 rpm) for 20 min at 4 °C; isopropanol contents were replaced with ethanol 70%. Next, they were centrifuged (15,000 rpm) for 5 min, discarded ethanol, and obtained plaques were dried at 40 °C. Finally, the plagues were dispersed in DEPC water, and the quality and quantity of the extracted RNAs were investigated using nanodrop apparatus (Synergy HTX Multi-Mode Reader, USA). The absorbance (OD) ratio at 260 nm and 280 nm was used to assess the purity of RNA and protein contamination. A ratio > 1.8 was accepted as the pure sample.

After that, cDNA was synthesized using the cDNA synthesis kit (Yectatajhiz, Iran). Briefly, the extracted total RNAs were mixed with oligo dT and DEPC water and incubated for 5 and 1 min at 70 °C and 4 °C. After that, 5X strand buffer, dNTPs (10 mM), RNasin (40 U/μL), and M-MLV were added into microtubes and subjected to the BIORAD Thermo cycler apparatus. The thermal program was set at 60 min at 42 °C, and obtained cDNAs were stored at − 20 °C.

The amplification was performed using RealQ Plus 2x Master Mix Green high ROX™ (Amplicon, Denmark) using the qPCR machine (StepOnePlus, Applied Biosystems, USA). Briefly, the mixture containing master mix (Green High Rox), forward and reverse primers for each gene (Pishgam Biotech Co., Tehran, Iran – Table 1), and cDNA template were adjusted to a final volume of 20 μL using DEPC water. Then, amplification reactions were carried out under the following conditions: 10 min at 95 °C, 40 cycles of 95 °C 15 sec, 55 °C 30 sec, and 72 °C 30 sec. In addition, relative fold changes in the expression of target genes (Bax and Bcl-2) with the β-actin gene as an internal control were normalized using the 2^˗ΔΔCT^ method [31], where ΔCT = CT target ˗ CT reference, ΔΔCT = ΔCT test sample ˗ ΔCT control sample, and relative expression = 2^˗ΔΔCT^.

**Table 1:** The primers sequences of genes

Gene name	Primer sequence
Bax	Forward: 5′- CCCGAGAGGTCTTTTTCCGAG - 3′
	Reverse: 5′ - CCAGCCCATGATGGTTCTGAT - 3′
Bcl-2	Forward: 5′ - GGTGGGGTCATGTGTGTGG - 3′
	Reverse: 5′ - CGGTTCAGGTACTCAGTCATCC - 3′
β-actin	Forward: 5′ - TCCTCCTGAGCGCAAGTAC - 3′
	Reverse: 5′ - CCTGCTTGCTGATCCACATCT - 3′

### Statistical analysis

The experiments were carried out in triplicates, and data are presented as mean ± standard deviation. One-way ANOVA followed by the Tukey Post Hoc test was used to compare samples (IBM SPSS statistics 22 software, Redmond, USA).

## Results

### MPEO analysis by GC-MS

Identified compounds in MPEO using GC-MS analysis are listed in Table 2. Pulegone (68.11%), l-menthone (8.83%), limonene (2.90%), iso-pulegone (2.69%), and iso-menthone (1.48%) were the five major ingredients.

**Table 2:** Identified compounds in MPEO by GC–MS

No.	Retention time (min)	Compound	%	Retention Index
1	11.39	α-Pinene	0.29	935
2	13.73	β-Pinene	0.20	982
3	14.90	3-Octanol	0.97	1005
4	16.44	Limonene	2.90	1035
5	23.21	L-Menthone	8.83	1169
6	23.63	Isomenthone	1.48	1177
7	24.25	Isopulegone	2.69	1190
8	27.45	Pulegone	68.11	1257
9	31.99	Piperitenone	1.10	1356
10	50.39	Myristic acid, isopropyl ester	0.22	1829

### Physicochemical properties of prepared nano-scaled emulsions and nanogel

**Fig. 1:**
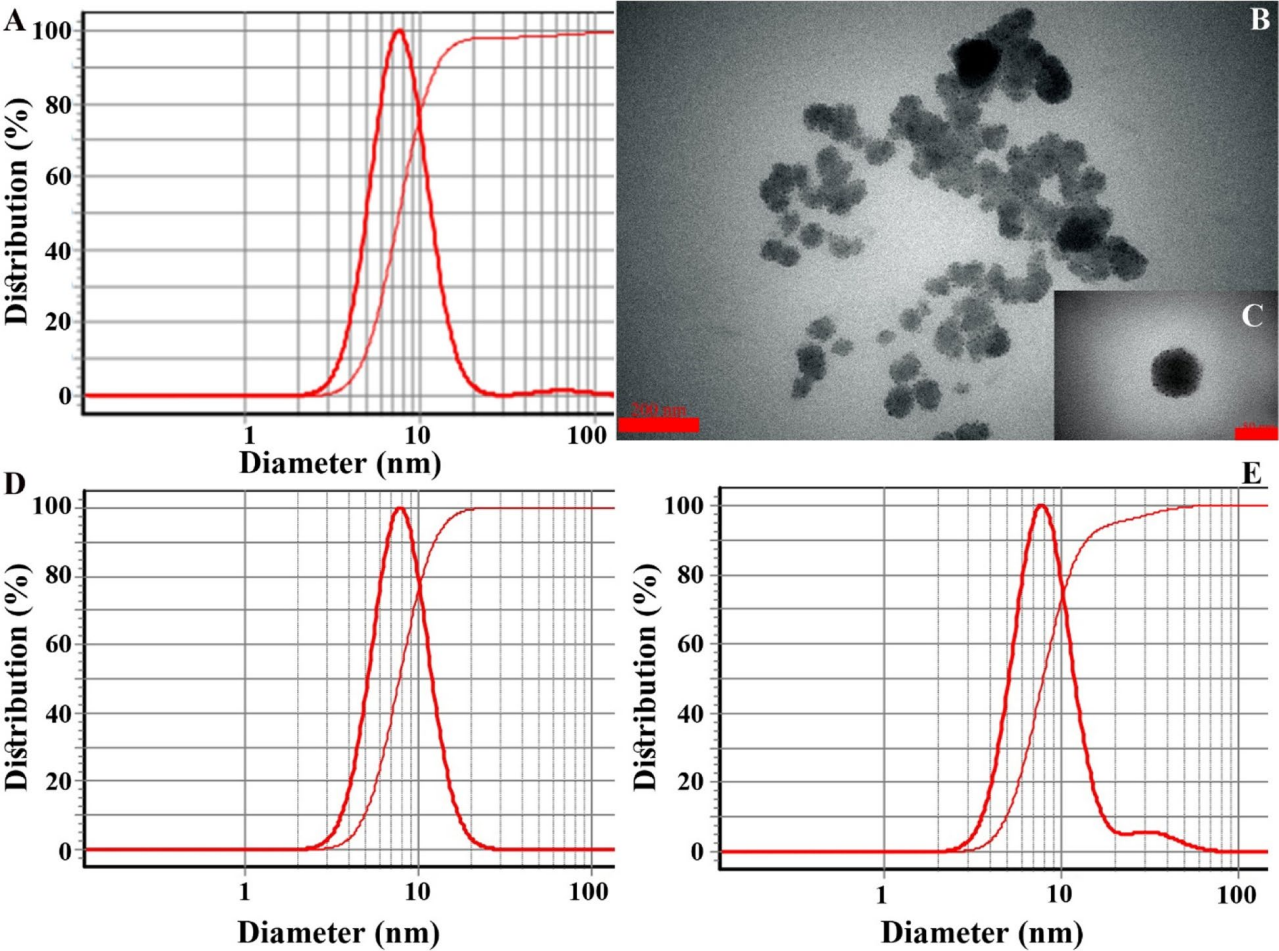
**A**: DLS profile of the selected nano-scaled emulsion with a droplet size of 7.70 nm; **B** and **C**: its TEM image with scale bars of 200 and 50 nm, **D**: its DLS profile after 60 min centrifugations (20,000 g) at −4, + 4, and 26 °C with a droplet size of 8.10 nm, **E**: its DLS profile after 9 months of storage at room temperature with a droplet size of 7.98 nm

Size analyses of the prepared nano-scaled emulsions and their ingredients are listed in Table 3. Among ten prepared formulations, only F8 with a droplet size of 7.70 nm and SPAN 0.99 possess proper size characteristics (Fig. 1A). It was selected for further investigations (biological assays) and gelation. TEM images of the selected nano-scaled emulsion are shown in Fig. 1 B and C; droplets are toothed sphere shape.

**Table 3:** Characteristics of the prepared nano-scaled emulsions containing MPEO

No.	Ingredients (μL)			Size analyses	
	MPEO	tween 20	PBS^**a**^	droplet size	SPAN^**b**^
F1	6	3	4991	495	1.81
F2	6	6	4988	29.2	19.69
F3	6	9	4985	127	1.58
F4	6	12	4982	9.48	15.45
F5	6	15	4979	7.70	21.48
F6	6	18	4976	6.69	3.17
F7	6	21	4973	6.90	2.96
F8	6	24	4970	7.70	0.99
F9	6	27	4967	6.69	2.36
F10	6	30	4964	7.35	1.30

^a^phosphate saline buffer

^b^droplet size distribution

The nano-scaled emulsion showed proper stability after stability analyses. Its DLS profile after 60 min centrifugation at − 4, + 4, and 26 °C is given in Fig. 1D; droplet size and SPAN were obtained at 8.10 nm and 0.96. Interestingly, due to the force applied in the centrifuge, the size of droplets had slightly increased by reducing droplet size distribution (i.e., SPAN). Besides, the DLS profile after 9 months of storage at room temperature is shown in Fig. 1E; droplet size and SPAN were obtained at 7.98 nm and 1.00. Moreover, no sedimentation and phase separation was observed after centrifugation and 9 months of storage stability tests.

**Fig. 2:**
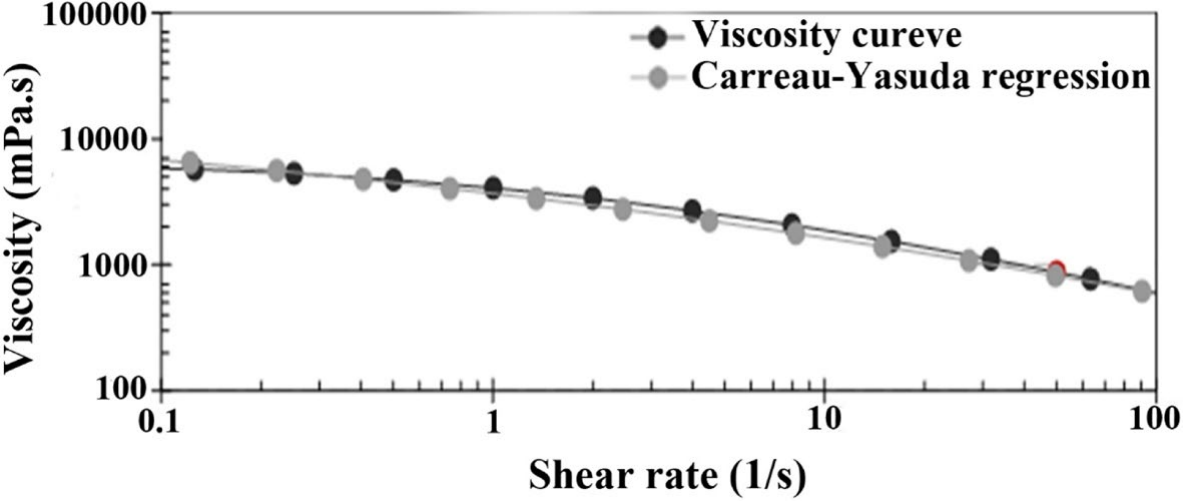
Viscosity curve of the nanogel

The nano-scaled emulsion was gellified by adding 2.0% w/v CMC; the viscosity of nanogel at different shear rates followed the Carreau-Yasuda model (see Fig. 2). The Carreau–Yasuda model suggests the dependency of shear complex viscosity on frequency [32]. Also, no phase separation, sedimentation, and creaming were observed in the nanogel after 6 months of storage at 4 °C and room temperature, which confirmed its proper stability.

**Fig. 3:**
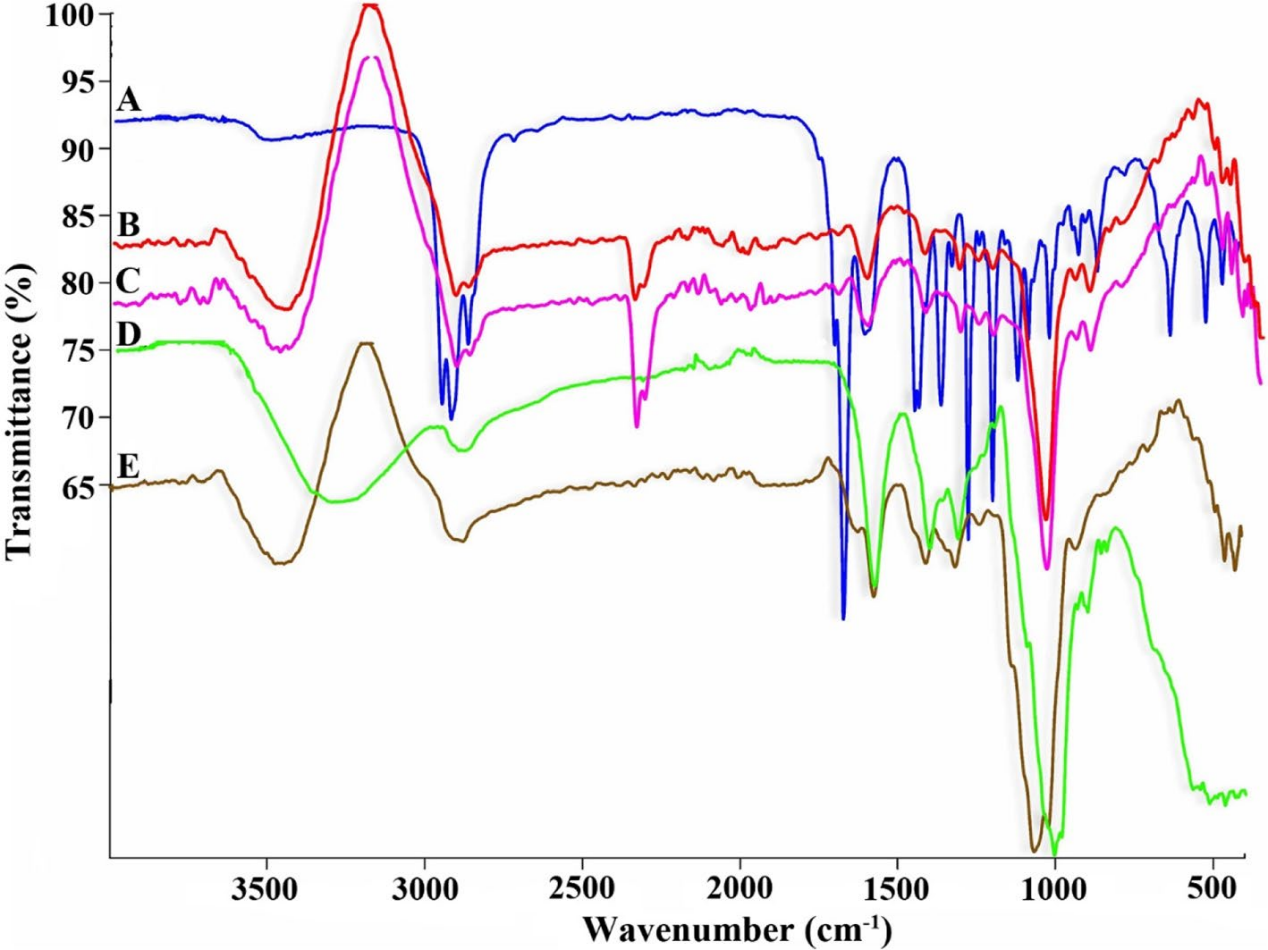
ATR-FTIR spectra of **A**: MPEO, **B**: blank emulsion, **C**: nano-scaled emulsion, **D**: CMC, **E**: nanogel

ATR-FTIR spectra of MPEO, blank emulsion, nano-scaled emulsion, CMC, and nanogel are illustrated in Fig. 3. ATR-FTIR spectrum of MPEO (Fig. 3 A) showed a broad band at about 3492 cm^− 1^ that can be related to the –OH stretching band. The peaks at 2963, 2924, and 2870 cm^− 1^ are attributed to C–H stretching vibration, the strong peak at about 1708 cm^− 1^ is related to the carbonyl group (C=O), and the bands at 1613–1454 cm^− 1^ are attributed to C = C stretching vibrations. The bands in the spectral region 1441 cm^− 1^ are due to CH2, and the band at about 1371 cm^− 1^ can correspond to -CH3 bending vibrations. The sharp peak at about 1281 cm^− 1^ corresponded to the C-O stretching vibration.

The spectrum of the blank emulsion (Fig. 3 B) displayed a broad peak at 3445 cm^− 1^, corresponding to OH stretching vibration because of the strong hydrogen bonding between water and tween 20. The band at 2920 cm^− 1^ is related to C-H stretching, the strong peak at 1639 cm^− 1^ showed carbonyl stretching (C=O), and the absorption band at about 1430 cm^− 1^ can be related to CH2 bending. The strong band at about 1083 cm^− 1^ corresponded to C-O stretching.

ATR-FTIR spectrum of the nano-scaled emulsion (Fig. 3 C) shows a band at about 3472 cm^− 1^ that can be related to OH stretching vibration. The peak at 2923 cm^− 1^ is related to C-H stretching in MPEO and tween 20. The strong band at 1730 cm^− 1^ is related to C=O stretching, representing the carbonyl group in MPEO, and the characteristic peak at about 1461 cm^− 1^ shows CH2 bending in MPEO and tween 20. Moreover, the characteristic and sharp peak at 1083 cm^− 1^ corresponds to C-O stretching.

The spectrum of CMC (Fig. 3 D) shows the broadband at about 3200–3500 cm^− 1^ related to hydrogen bonding between –OH groups. Furthermore, the characteristic band at 2894 cm^− 1^ corresponds to C–H stretching vibration, and the peaks at about 1587 and 1411 cm^− 1^ displayed carboxylate groups stretching vibrations (symmetric and asymmetric). The strong peak at about 1014 cm^− 1^ can be related to C-O stretching.

ATR-FTIR spectrum of the nanogel (Fig. 3 E) shows the broadband at about 3453 cm^− 1^ related to –OH stretching vibration. The band at 2886 cm^− 1^ is assigned to C-H stretching due to MPEO, tween 20, and CMC. The strong peak at 1583 cm^− 1^ exhibited COO stretching in tween 20. The sharp peak at 1074 cm^− 1^ corresponds to C-O stretching. The COO- band at 1587 cm^− 1^ in the presence of CMC was shifted toward the lower wave number at 1583 cm^− 1^, confirming the association of CMC with tween 20 through intermolecular H-bonding. The spectra of nanogel show that the band strength is significantly wider and shifted higher than 3340 cm^− 1^ (in CMC), attributed to the stretching vibration of CMC, hydroxyl group in tween 20, and MPEO. The degree of bond polarization is significantly increased with the formation of hydrogen bonds. Physical cross-linking between the hydroxyl groups of CMC, tween, and MPEO also consumes little hydroxyl groups.

### The cytotoxicity effects of samples

**Fig. 4:**
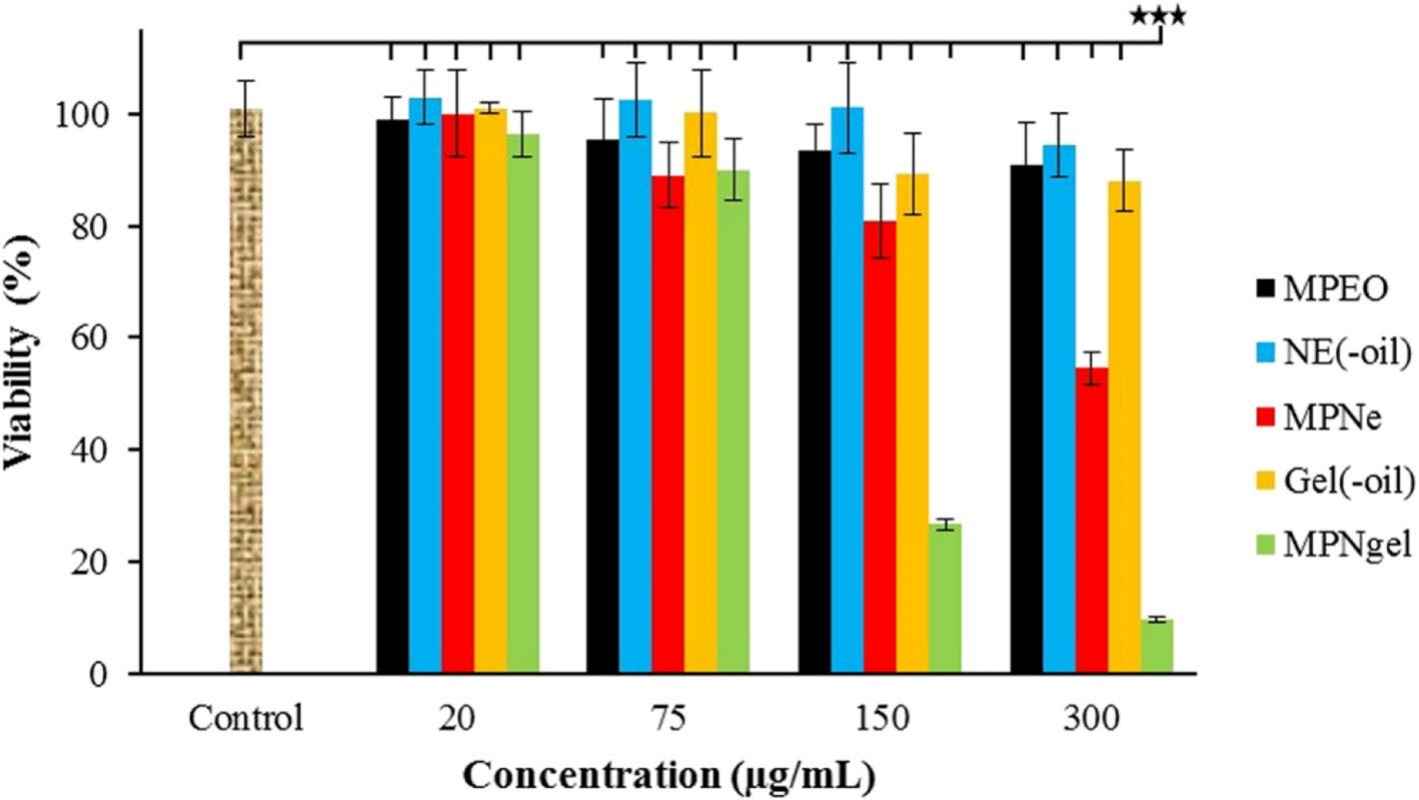
Cytotoxicity effect of MPEO, blank emulsion (NE(−oil)), nano-scaled emulsion (MPNe), blank nanogel (Gel(−oil)), and nanogel (MPNgel) on A375 cells. *** *P* < 0.001

The cytotoxicity of different concentrations of MPEO, blank emulsion, nano-scaled emulsion, blank nanogel, and nanogel against the A375 cells are shown in Fig. 4. MPEO and blank emulsion did not show important effects on cell viability (< 10%). Besides, Gel(−oil) showed slight toxicity towards cells at concentrations of 150 and 300 μg/mL; viabilities decreased by about 12%. However, the viability of cells decreased in a dose-dependent manner after treatment with nano-scaled emulsion and nanogel. The nanogel 300 μg/mL showed substantially (*P* < 0.001) more potency than other samples; viability decreased by more than 90%. Moreover, the viability of cells decreased by 45% after treatment with nano-scaled emulsion 300 μg/mL.

### Apoptosis detected using flow cytometry

**Fig. 5:**
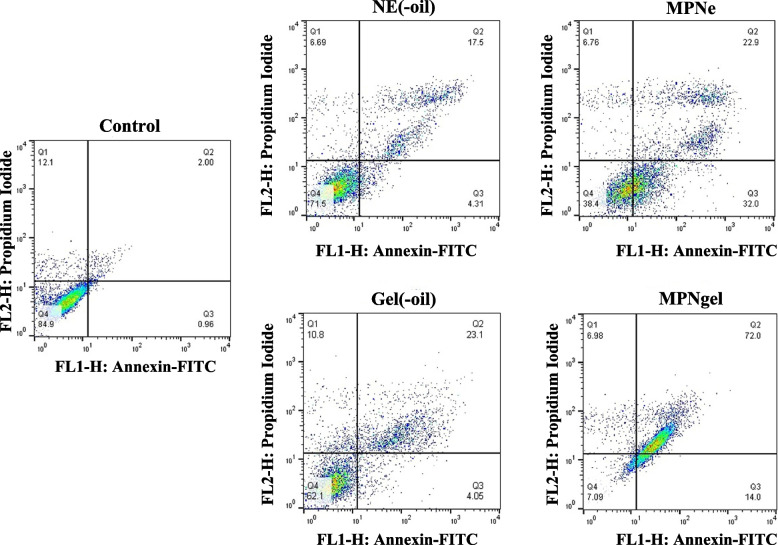
Flow cytometry analysis of apoptosis in A375 cells treated (24 h) with blank emulsion (NE(−oil), nano-scaled emulsion (MPNe), blank nanogel (Gel(−oil)), and nanogel of MPEO (MPNgel). The histograms show 2 ± 0.5% of cells at late apoptotic (Annexin V + PI+) in control, 17.5 ± 2% in NE(−oil), 22.9 ± 2.5% in MPNe, 23.1 ± 1.5% in Gel(−oil), and 72.0% ± 4% in MPNgel treated cells

Figure 5 shows the population of viable (Annexin V - PI-), necrotic (Annexin V- PI+), early apoptotic (Annexin V + PI-), and late apoptotic (Annexin V + PI+) cells. The nanogel induced early apoptosis by 14.00% and late apoptosis by 72% compared to the control group (0.96 and 2%). Moreover, nano-scaled emulsion induced early apoptosis by 32.00% compared to control, blank nanogel, blank emulsion, and MPNgel at 0.96, 4.05, 4.31, and 14.00%, respectively.

### Effect of samples on apoptosis regulatory genes expression (Bax and Bcl-2)

**Fig. 6:**
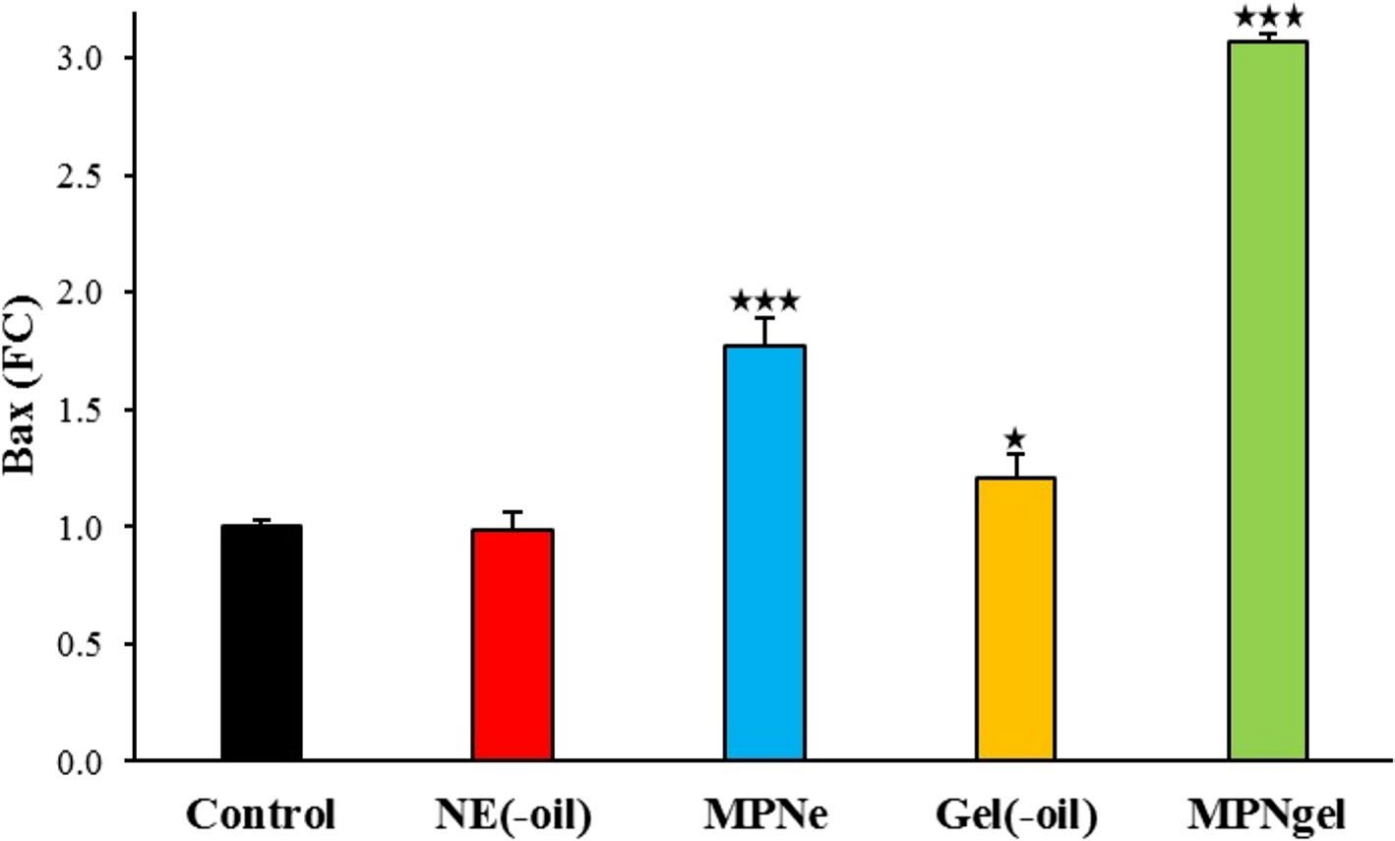
Bax gene expression in the A375 cell line after treatment with blank emulsion (NE(−oil), nano-scaled emulsion (MPNe), blank nanogel (Gel(−oil), and nanogel (MPNgel) compared to the control group (untreated). *P < 0.05, **P < 0.01, and ***P < 0.001

**Fig. 7:**
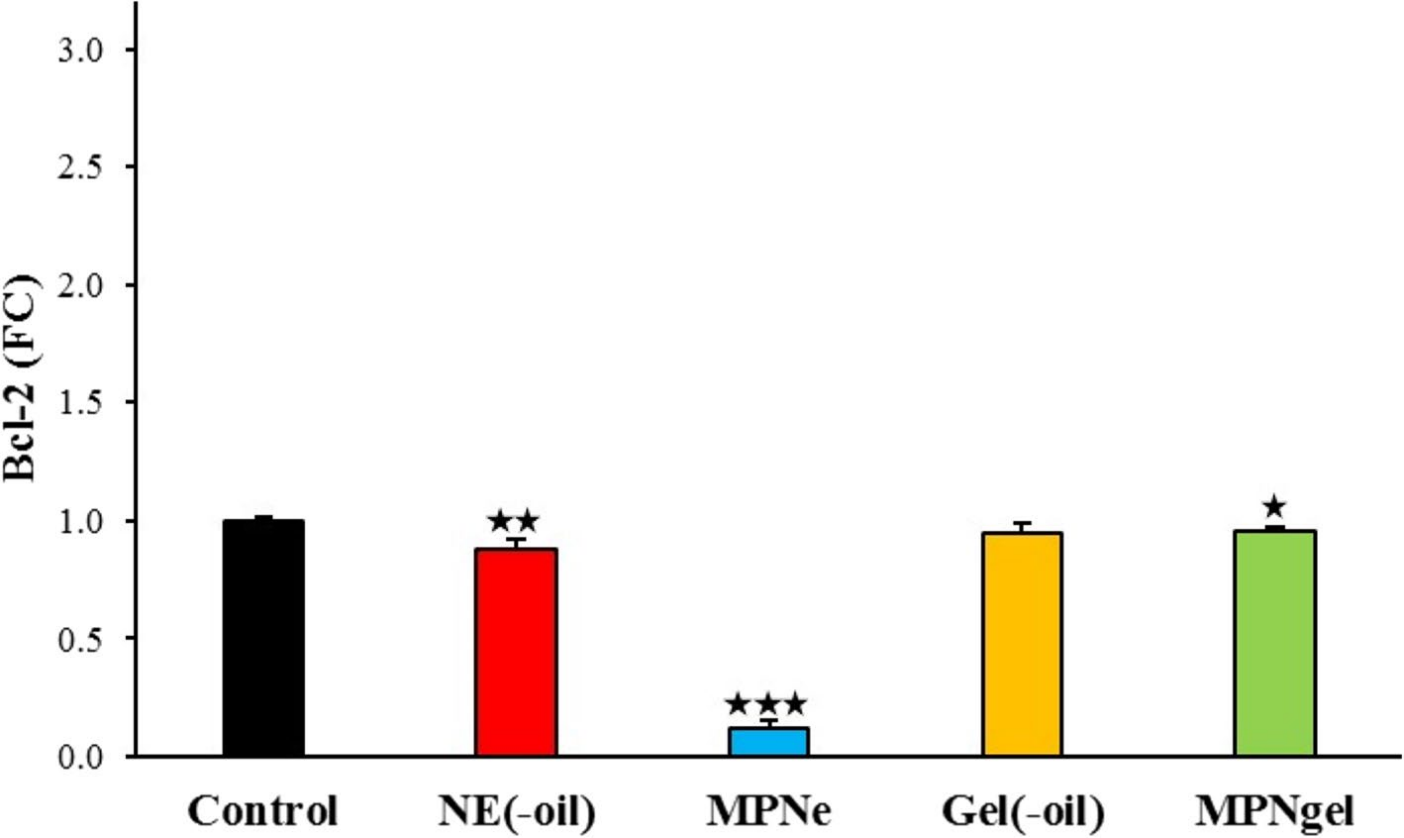
Bcl-2 gene expression in the A375 cell line after treatment with blank emulsion (NE(−oil), nano-scaled emulsion (MPNe), blank nanogel (Gel(−oil), and nanogel (MPNgel) compared to the control group (untreated). *P < 0.05, **P < 0.01, and ***P < 0.001

The Bax and Bcl-2 gene expression are shown in Figs. 6 and 7. A significant increase in the expression of the pro-apoptotic gene (Bax) was observed after treatment with nano-scaled emulsion (*P* < 0.001), blank nanogel (*P* < 0.029), and nanogel (*P* < 0.001) compared to control group. Besides, a significant decrease in the expression of the anti-apoptotic gene (Bcl-2) was observed after treatment with blank emulsion (*P* < 0.009), nano-scaled emulsion (*P* < 0.001), and nanogel (*P* < 0.037) compared to the control group. Overall, results indicate the potential efficacy of nanogel and nano-scaled emulsion in directing cancer cells toward the apoptosis pathway.

## Discussions

Due to the side effects of chemotherapeutic drugs, investigating the anticancer effects of EOs has been a hot field in recent years. EOs and their components, especially terpenes, possess a wide range of biological effects such as antibacterial, antioxidant, and anticancer [33, 34]. For example, *Schefflera heptaphylla* EO with β-pinene as the major compound showed antiproliferative activity against the A375 cells with IC50 values of 198.5 and 264.7 μg/mL [35]. Besides, *Afrostyrax lepidophyllus* and *Scorodophloeus zenkeri* EOs showed a promising growth-inhibitory effect on the A375 cells with IC50 values of 20.6 and 17.7 μg/mL [36]. This study used MPEO as a natural therapeutic agent; an attempt was made to improve its cytotoxicity against A375 cells by preparing nano-scaled emulsion and nanogel dosage forms. Interestingly, a substantial improvement was observed; cell viability was observed at 91, 55, and 9% after treatment with 300 μg/mL of MPEO, nano-scaled emulsion, and nanogel. Significant improvement in efficacy after nanoformulation of EOs has been mentioned in published reports. For instance, the viability of A375 cells after treatment with 300 μg/mL *M. longifolia* EO and solid lipid nanoparticles containing *M. longifolia* EO were observed at 95 and 27% [18]. Also, A375 cell viability after treatment with 250 μg/mL *Myrtus communis* EO and its nanogel dosage form at 70 and 17% [37]. Moreover, after treatment of A375 cells with 300 μg/mL of *M. longifolia* EO and chitosan nanoparticles containing the EO viability were reported at 72 and 31% [38].

Nano-scaled emulsions are emulsions with droplet sizes on the nanometer scale. In addition to biocompatibility and biodegradability, straightforward preparation methods, improved absorption, and increased drug bioavailability are the main advantages of nanoemulsions [39]. Furthermore, the ability to hydrate the skin, lack of skin irritation, and high drug-loading capacity are some interesting properties of nanoemulsions in topical drug delivery [40, 41]. However, low viscosity is their only challenge for topical drug delivery. Nanoemulsion-based nanogels have thus been introduced as a promising dosage form in topical drug delivery; they exploit the advantages of nanoemulsion and improve their viscosity and stability [42, 43]. Nowadays, nanogels have been widely used to improve the topical delivery of hydrophobic drugs. For instance, a nanoemulsion-based nanogel containing amphotericin B was prepared using carbomer 980. Its percutaneous permeation flux rate of nanogel (18 μg/cm2/h) was better than nanoemulsion (16 μg/cm2/h) and drug solution (5 μg/cm2/h) [44]. This study used CMC as the gelling agent, a semi-synthetic anionic polymer. Its distinct properties, such as mechanical resistance, low cost, and proper stability, make it an ideal gelling agent in developing topical drug delivery systems [45, 46]. For instance, chitosan-CMC nanogel containing *Nigella sativa* EO and atorvastatin were used to treat skin lesions [47]. Another study showed that CMC nanogel containing curcumin was an ideal option for treating melanoma skin cancer (MEL-39) [48].

One of the earliest apoptotic features is the translocation of phosphatidylserine from the inner to the outer layer of the membrane. The Annexin-V/FITC binding assay is a reliable technique to detect apoptosis by flow cytometry; the exact percentage of apoptotic cell death is determined [49]. In the current study, Annexin-V/FITC/PI was used to determine whether cells are destroyed by apoptosis or decomposition due to environmental stresses and necrosis. The results showed that MPNgel had the highest cell death percentage due to apoptosis.

Furthermore, tumor cells promote survival by increasing Bcl-2 anti-apoptotic proteins and decreasing apoptotic proteins such as Bax. Also, Bcl-2 interacting with Bax as apoptotic-promoting gen inhibits apoptosis in cancer cells [50, 51]. Moreover, changes in the expression level of Bax and Bcl-2 cause mitochondrial instability, leading to the release of apoptotic-inducing factors. The present study showed a significant difference in the expression of these two genes in treated cells with the nanogel and nano-scaled emulsion compared to the control group. Considering flow cytometry and qPCR results, it could be concluded that cells entered programmed death, and the expression of apoptotic genes was changed accordingly.

## Conclusion

The chemical composition of *M. pulegium* EO was identified. A comprehensive comparison was then carried out between the cytotoxic effect of the EO and its nano-scaled emulsion and nanogel dosage form against A375 cells. The efficacy of nanogel was significantly more potent than other samples. Furthermore, flow cytometry and qPCR results confirm that the samples induce cell death via the apoptotic pathway. Therefore, given the proper potency of nanogel, it could be considered for further in vivo investigation.

## Data Availability

All data are available from corresponding authors on reasonable request.
